# Smart Pacemaker: A Review

**DOI:** 10.7759/cureus.30027

**Published:** 2022-10-07

**Authors:** Shivi Agarwal, Raju K Shinde

**Affiliations:** 1 Otolaryngology, Jawaharlal Nehru Medical College, Datta Meghe Institute of Medical Sciences, Wardha, IND; 2 General Surgery, Jawaharlal Nehru Medical College, Datta Meghe Institute of Medical Sciences, Wardha, IND

**Keywords:** dyssynchrony, cardiac resynchronization treatment, arrhythmias, biomedical electronic implant, electronic pacemaker

## Abstract

Since the first pacemaker was implanted, nearly 60 years have passed. Since then, pacemaker technology has made major advancements that have increased both its safety and effectiveness in treating people with bradyarrhythmias. The repeated stimulation of cells in specialized "pacemaker" regions of the mammalian heart and the transmission of stimulus via the ventricles serve as evidence that the electrical function of the mammalian heart is necessary for a regular mechanical (pump) role. The development of action potentials in individual cardiac cells is linked to myocardial electrical activity and the heart's regular cooperative electrical functioning. A container or pulse initiator that houses the battery and electronics, as well as lines that connect to the myocardium to deliver a depolarizing pulse and detect intrinsic cardiac stimulation, are all parts of a pacemaker. Defibrillators could be used with artificial hearts that have electrical pacemakers integrated into them in order to treat arrhythmia, heart failure, and cardiac arrest. Modern pacemakers have units for supporting patients with other disorders like "heart failure," which happens when the heart does not pump as forcefully as it should. While many pacemakers are effective in treating different types of arrhythmias (irregular heartbeats), they also have units for treating them.

## Introduction and background

Despite significant advancements and the development of novel therapies, cardiovascular illnesses have remained the world's leading cause of death and morbidity for a decade [1]. Several treatments, including cell-based therapies, have been shouldered in the last 20 years; however, penurious subsister and injection of relocated cells in the ischemic environment of cardiac tissue have restricted their clinical usefulness [2]. One of the most pressing issues in the industry is the capacity to track the fate of modified tissue and its impact on the nursed organ after transplantation. Furthermore, when it is feasible to observe the activity of the implanted tissue, a tool to intercede in the therapy's outcome without the obligation for additional surgical mediation or ongoing medical attentiveness would prove to be highly advantageous to the therapy's effectiveness [3,4].

It has been almost six decades since the initial pacemaker was placed. Since then, pacemaker technology has advanced significantly, boosting its safety and potency in treating individuals with bradyarrhythmias. Despite advancements, pacemaker therapy continues to be analogous with considerable peri- and post-procedural problems [5].

Leadless pacemaker therapy is a revolutionary method that addresses lead and pocket-associated issues in standard transvenous pacemaker treatment and was recently launched in clinical trials. These leadless pacemakers are independent, single-chamber suitable ventricular pacemakers inserted by a femoral percutaneous route [6].

## Review

Physiology 

The heart is a metrical electromechanical pump whose operation relies on the origination and transmission of action potentials, tailed by relaxation and a phase of refractoriness until a succeeding stimulus is produced. Inward (Na+ and Ca2+) and outward (K+) impulse-transporting ion channels are consecutively switched on and off during myocardial action potentials. Action potential waveforms vary in separate parts of the heart due to variances in Na+, Ca2+, and K+ channel articulation. These variations add up to a typical, unidirectional impulse circulation and the creation of usual cardiac rhythms [7].

The electrical function of the mammalian heart is required for a regular mechanical (pump) role, as evidenced by the successive stimulation of cells in specialized "pacemaker" areas of the heart and the transmission of stimulus via the ventricles. The creation of action potentials in discrete cardiac cells is attributed to myocardial electrical venture, and normal cooperated electrical working of the whole heart. Changes in channel function caused by inherited or acquired illness impact the action potential repolarization and can result in life-threatening arrhythmias [7].

Tissue engineering 

When the heart cannot keep up, technology comes up with a solution: an artificial pacemaker, a medical apparatus that provides electrical activity to keep the heartbeat stable. Artificial pacemakers have been used to treat various heart diseases that cause them to beat abnormally since the late 1950s, and they come with varying degrees of programmability.

Pacemakers comprise a pulse initiator or can accommodate the battery and electronics, as well as lines that pass from the can to the myocardium to impart a depolarizing pulse and ascertain intrinsic cardiac stimulus. Insulation materials separate the conductor cables and the lead tip electrodes. The leads might be concentric (a tube within a tube) or co-radial, depending on the relationship between the wires (side-by-side coils). Active (with an electrically active helix at its tip for mechanical strength) or passive lead attachment to the myocardium (electrically inert tines harbor the lead). Short-circuiting causes high impedance (fracture) or low impedance (insulation breach) depending on how conductor elements and insulation materials are disrupted. When a potential difference (voltage) is enforced between the two electrodes, the pacing starts to ensue [8,9].

The devices are made entirely of FDA-approved biocompatible materials; nevertheless, they are non-biodegradable and will persist in the patient's body for the rest of their lives until they are removed. Although not always harmful, there are times when the existence of old-fashioned electrical implants within the tissue is dispensable. If these non-biodegradable devices become a regular place, they could represent a danger to the patient's life [3,10].

Pacemakers today use activity sensors (also known as accelerometers) to measure the patient's movement. However, these sensors are frequently insufficient to deliver real-time automated heart rate variation to the wearer. It might fluctuate depending on the rate of respiration; for example, current pacemakers are not sensitive enough to monitor and process specific brain activities related to heart rate control. As a result, even if the patient is cycling, the pacemaker indicates that they are at rest since precise signals are not refined. When a neuronal circuit at the base of the brain depreciates due to age or disease, it fails to provide the correct signals for the heart to pump correctly [11].

Hybrid technique 

Substituted hearts with built-in electrical pacemakers could be employed as a defibrillator in the event of arrhythmia or failure, as well as in the event of cardiac arrest. Of course, they would be merged accompanied by a network of electrodes that can provide a complete account of the organ's condition [3,12].

**Figure 1:**
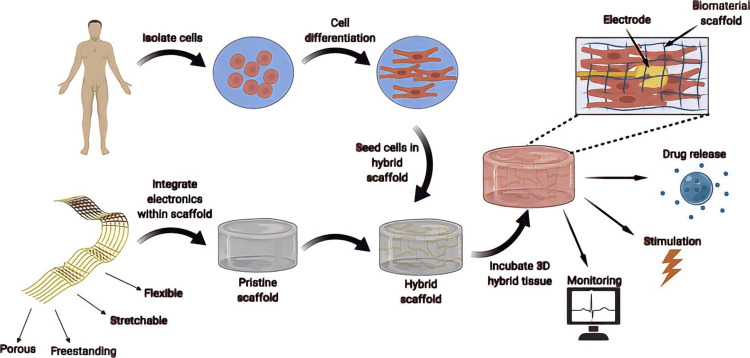
Hybrid technique [3]

Tian and colleagues provided the first example of a hybrid technique. A planar, slim, penetrable, and electronic mesh with amalgamated silicon nanowire field-effect transistors was created via lithography (nanoFETs). By removing an underlying sacrificial layer, this apparatus was subsequently removed from the silicon wafer and transformed into a discrete device. The mesh was designed to be highly penetrable, and its thin thickness of 2mm made it incredibly pliable and convenient to handle in 3D as shown in Figure 1. Afterward, the nanoelectronic webbing was embedded in a leveled electrospun biomaterial fiber mat and employed as a hybrid setting for sowed neurons and cardiomyocytes because the hybrid material is so pliable that it might be rolled and folded into a broader 3D-designed tissue following cell seeding. The research proved the production of engineered cardiac tissue in which the electronics had a negligible impact on its organization, as well as the scaffold's ability to observe the tissue's function from within a spatiotemporal manner [13,14].

Two present-day examples have revealed the use of electrospun fiber scaffolds as the membrane and dielectric of the electronic net in order to construct a hybrid tissue made of biodegradable electronics [7]. The synthetic polymer polyurethane was utilized in one example (Lee et al. 2019) [15], whereas the innate protein albumin was used in the other (Feiner et al., 2018) [16]. Both studies used biodegradable substances that can be used as a podium for tissue engineering and can be electrospun into fiber configuration to create flexible and elastic scaffolds. It was viable to build a mesh that functions as an electronic unit and a scaffold for modified tissue by evaporating metal electrodes onto electrospun fiber scaffolds through a shadow mask. Cardiomyocytes planted on these pieces of equipment are organized into utilitarian heart tissue just like their perfect counterparts. It was feasible to document extracellular potentials from the confines of the created tissues, activate them and deliver anti-inflammatory medications in a regulated approach using evaporated gold electrodes [3,17,18].

The Micra Transcatheter Pacing System and the Nanostim Leadless Cardiac Pacemaker (L.C.P.; St. Jude Medical) are now leadless pacing systems in clinical use (T.P. S., Medtronic). Both the devices are self-sufficient and can single chamber pace sensing, and rate reaction supply The Nanostim L.C.P got the C.E. mark in October 2013 [6]. However, Micra Transcatheter Pacing System now comes in an AV version with dual chamber sensing and RV pacing. The Nanotism Leadless Cardiac Pacemaker has been replaced by the AVIRA Leadless system. 

Cardiac resynchronization therapy

**Figure 2:**
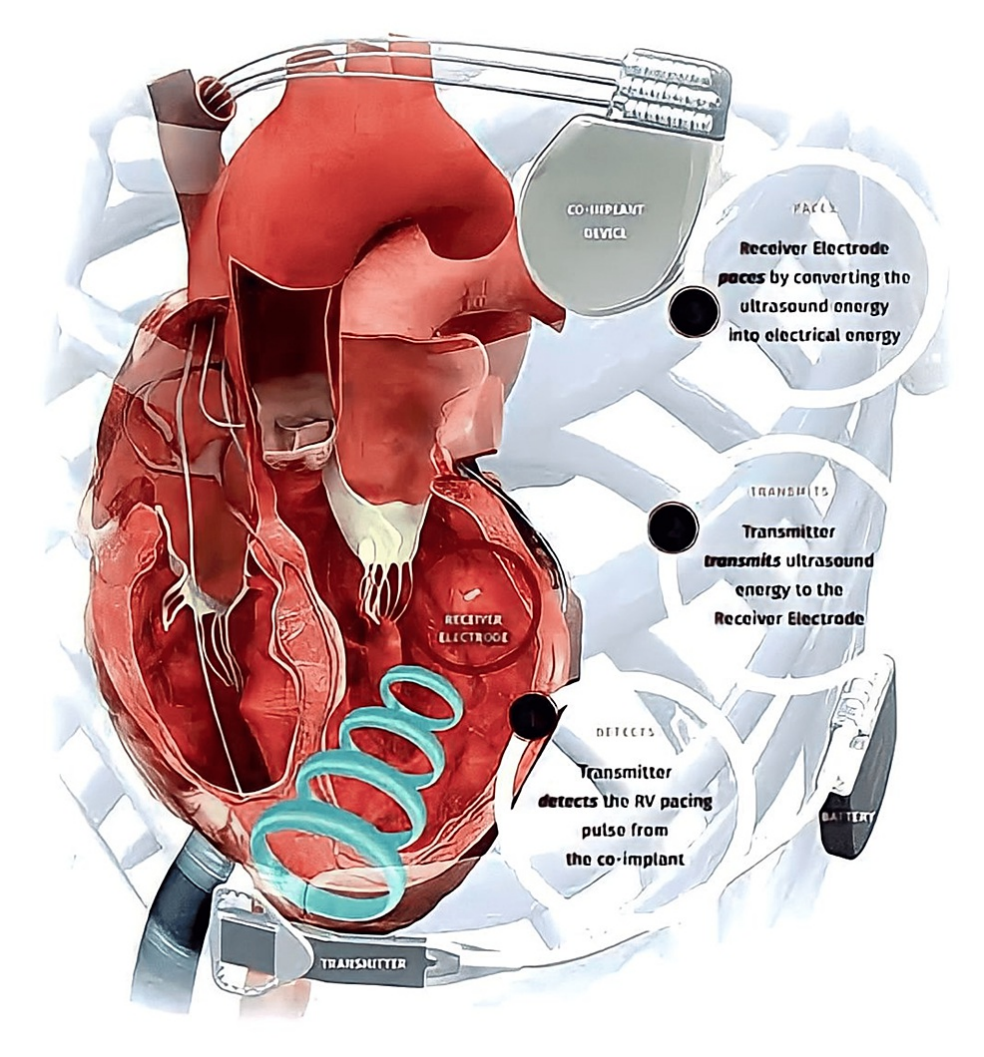
Working of CRT [22]

In patients with left ventricular (LV) dysfunction, choosing the suitable implanted cardioverter defibrillator (ICD) or cardiac resynchronization treatment (CRT) device might be difficult [19]. ACC/AHA Guidelines for conduction system disorders and another set of ACC/AHA Guidelines for the management of patients with ventricular arrhythmia can assist doctors in order to select the best gadget for the patient. Certain patients gain from cardiac resynchronization therapy (CRT) with advanced congestive heart failure. The Left Ventricular (LV) Pacing Lead Implantation Procedure Using the Overlay Ref Technique may be facilitated [20]. A depiction of CRT is shown in Figure 2. An innovative electromagnetic navigation system that shows the real-time 3D location of delivery instruments with implanted sensors circumfused on previously recorded X-ray cine-loops of coronary sinus venograms has been created to aid in the installation of left ventricular (LV) leads. When the new guiding system is used, it is possible to implant CRTs safely, successfully, and with substantially less radiation exposure [21].

Despite the promising results of CRT in bradycardia patients, not all patients experiencing RV pacing develop LV dysfunction; some may be impervious to the pacing-induced systolic dyssynchrony. Because CRT is expensive and has a high rate of complications, not every patient should receive it. It is crucial to pick people susceptible to systolic dyssynchrony from repeated ventricular pacing in bradycardia. Even though it might be ideal to find baseline indicators of pacing-induced systolic dyssynchrony, there is not enough information to guide patient selection [23]. Another recent problem is the CRT-induced pro-arrhythmia that may be connected to the LV lead inside the epicardial scar. It is an uncommon but significant consequence that antiarrhythmic medications cannot treat. A clinical conundrum arises when LV pacing is turned off because HF may deteriorate. Catheter ablation can treat recurrent arrhythmias; however, patients still need additional treatment. According to clinical data, women benefit from CRT more than males, but fewer women compared to men participated in the CRT clinical trials. Increased hiring of female prospects might lower the rate of non-responders. Although CRT is not advised for patients with narrow QRS, one of the research of discrete patient data found that females showed favorable results with CRT-defibrillator at a lower QRS length than males, which emphasizes the need for gender-specific medicine [24].

CRT is a costly therapy, which makes reimbursement difficult [25]. The relatively high complication rate associated with CRT as a result of the intricate structure of the coronary vein is another problem that affects its utilization in clinical practice. Compared to ICD or RV pacing, implanting takes more knowledge, expertise, and training. Lead displacement and dislodgement, implantation dissection, as well as phrenic nerve activation are risks connected with the insertion of CRT It is not surprising that the benefits of CRT have been disputed during its 20-year history, and its long-term effectiveness has been questioned. Despite having clear indications for CRT, a significant portion of patients received ICDs. Additionally, almost one-fourth of HF patients had RV pacing with frequent ventricular pacing percentage installed. More instruction with medical care based on guidelines is necessary for both patient and physician groups to address the low participation rate.

CRT is now used in patients with congenital heart disease with various HF phenotypes (systemic LV failure, RV failure, and single ventricle failure), hypertrophic cardiomyopathy, pulmonary hypertension causing RV failure, and HF with intact ejection fraction [23]. A multifaceted therapy proposition, such as AV junction ablation or pulmonary vein isolation in conjunction with CRT, might enhance the feedback to CRT with the aid of rate control because the efficacy of CRT is diminished in atrial fibrillation. Creating new technology is necessary to overcome barriers that prevent CRT from being widely used. The LV lead access route, such as the percutaneous subxiphoid approach or transventricular passage, must be improved in challenging patients. It has been shown safe and effective to implant CRT with the aid of an electromagnetic tracking system based on sensors [26].

Complications 

Since the transcatheter aortic valve replacement (TAVR) technology is applied to healthier and lower-risk groups, the implications and anticipations of procedure-related problems, together with the requirement of permanent pacemaker (PPM) installation, need to be determined [27]. Due to the modification of a conduction system that is already ill, researchers have revealed a greater prevalence [28,29].

All patients had paravalvular aortic regurgitation after PHV implantation. Echocardiography suggested that the PHV stent frame may not be ideally positioned against the sick native valvular structures in the area of calcific nodules. Even though the initial improvement in left ventricular function and clinical status following consolation of the aortic valve blockage was unaffected by paravalvular aortic regurgitation, severe paravalvular aortic regurgitation may have a deteriorating effect on long-term clinical outcomes succeeding PHV implantation. Future developments in stent design, such as larger maximal stent diameters, may lessen the frequency and graveness of paravalvular aortic regurgitation [30,31].

Other crucial factors to take into account with any valve-replacement procedure include issues such as postoperative stroke, valve degeneration, PVL, and conduction abnormalities. With combined rates of 2.7 percent at 30 days and 4.8 percent at prolonged follow-up, the occurrence of postoperative neurological events was less and appeared to correspond to the previously described prevalence of strokes for traditional AVR [32]. To more fully evaluate the protectiveness and effectiveness of SURD-AVR in comparison to the previously mentioned options, more trials comparing SURD-AVR to traditional AVR and TAVR with lasting results, a minimum deficit of follow-up, and randomization are required.

Future prospects 

Although there have been considerable advancements in the domain, each variety of tissue under investigation has its own set of hurdles that must be addressed before it can be used in clinical settings. The idea of incorporating electronics into all types of synthetic tissues has a lot of potential for improving tissue development and function [3,33].

While many modern pacemakers are effective in treating various types of arrhythmias (irregular heartbeats), they have units for supporting patients with other disorders such as "heart failure", which occurs when the heart does not pump as powerfully as it should [34,35].

In pacemaker (PM) patients, high abidance to remote monitoring (RM) improves outcomes; yet adherence remains unsatisfactory without a bedside console newer-generation PMs using Bluetooth low-energy (BLE) technology can communicate straightaway with patient-controlled intelligent devices via an app [36,37]. 

A smart pacemaker is a sophisticated device that can detect even the smallest irregularity in the functioning of the heart. These smart pacemakers then not only help in rectifying the abnormality in the heart and reestablishing normal body functions but also alert the patients and their physicians about the patient's cardiac health. Hence, allowing them to take appropriate measures for the same. This feature of smart pacemakers had helped bring down the mortality rate in today's world.

## Conclusions

The sector of biomedical electronic installs has progressed from the times of rigid implantable pacemakers to micro- and nanoscale, delicate electronic webs having traits as small as single cells and mechanical characteristics compared to the softest tissues. We have been able to bypass the considerable organic-inorganic hurdle that customarily subsists in joining electronics, tissues, and organs thanks to rapid breakthroughs in the sphere of flexible and stretchable electronics. These advancements have made it possible to counteract the detrimental effects that a foreign implant may have on a tissue and its denial by the body. The electronics could be removed after the organ function has been reinstated, and there is no obligation for ongoing inspection or mediation. On the other hand, if the existence of the electronics does not impair tissue behavior, they may be left in place indefinitely to ameliorate the patient's standard of life. The evolution of these sorts of substitutions would not only assist minimized donor organ scarcity but would also lessen the necessity for persistent follow-ups and operations. For carefully chosen individuals with heart failure, cardiac resynchronization treatment (CRT) is a beneficial therapeutic. Recent research indicates that re-coordinating left ventricular dyssynchrony may not be how CRT delivers the majority, if not all, of its advantages. Other probable mechanisms of effect that may differ from patient to patient and over time include atrioventricular resynchronization, decreased mitral regurgitation, and avoidance of bradycardia. No one treatment target exists; hence it is unlikely that any one metric will be able to predict benefit with any degree of accuracy. The purpose of this article was to create awareness among the medical society about smart pacemakers.

## References

[1] Roth GA, Johnson C, Abajobir A (2017). Global, regional, and national burden of cardiovascular diseases for 10 causes, 1990 to 2015. J Am Coll Cardiol.

[2] Passier R, van Laake LW, Mummery CL (2008). Stem-cell-based therapy and lessons from the heart. Nature.

[3] Feiner R, Dvir T (2020). Engineering smart hybrid tissues with built-in electronics. iScience.

[4] Smulyan H (2019). The computerized ECG: Friend and foe. Am J Med.

[5] Clémenty N, Fernandes J, Carion PL (2019). Pacemaker complications and costs: a nationwide economic study. J Med Econ.

[6] Tjong FV, Reddy VY (2017). Permanent leadless cardiac pacemaker therapy: A comprehensive review. Circulation.

[7] Nerbonne JM, Kass RS (2005). Molecular physiology of cardiac repolarization. Physiol Rev.

[8] Mulpuru SK, Madhavan M, McLeod CJ, Cha YM, Friedman PA (2017). Cardiac pacemakers: Function, troubleshooting, and management: Part 1 of a 2-part series. J Am Coll Cardiol.

[9] Groeneveld SA, Jongejan N, de Winter BJSAAF, Fiolet ATL (2019). [Hacking into a pacemaker; risks of smart healthcare devices]. Ned Tijdschr Geneeskd.

[10] Ostiguy G, Black T, Bluteau LJ (2013). Smart meters and routers radiofrequency disturbances study with pacemakers and implantable cardiac defibrillators. Pacing Clin Electrophysiol.

[11] Brown JM, O'Brien SM, Wu C, Sikora JA, Griffith BP, Gammie JS (2009). Isolated aortic valve replacement in North America comprising 108,687 patients in 10 years: changes in risks, valve types, and outcomes in the Society of Thoracic Surgeons National Database. J Thorac Cardiovasc Surg.

[12] Tian B, Liu J, Dvir T (2012). Macroporous nanowire nanoelectronic scaffolds for synthetic tissues. Nat Mater.

[13] Padeletti L, Barold SS (2005). Digital technology for cardiac pacing. Am J Cardiol.

[14] Klonoff DC (2011). Smart sensors for maintaining physiologic homeostasis. J Diabetes Sci Technol.

[15] Lee S, Sasaki D, Kim D (2019). Ultrasoft electronics to monitor dynamically pulsing cardiomyocytes. Nat Nanotechnol.

[16] Feiner R, Fleischer S, Shapira A, Kalish O, Dvir T (2018). Multifunctional degradable electronic scaffolds for cardiac tissue engineering. J Control Release.

[17] Yaksh A, van der Does LJ, Kik C (2015). A novel intra-operative, high-resolution atrial mapping approach. J Interv Card Electrophysiol.

[18] Joury A, Bob-Manuel T, Sanchez A (2021). Leadless and wireless cardiac devices: The next frontier in remote patient monitoring. Curr Probl Cardiol.

[19] Curcio A, DE Rosa S, Sabatino J (2016). Clinical usefulness of a mobile application for the appropriate selection of the antiarrhythmic device in heart failure. Pacing Clin Electrophysiol.

[20] Zhang BC, Tang K, Xu YW (2011). Initial clinical experience with implantation of left ventricular lead guided by Overlay Ref for the treatment of congestive heart failure. Arch Cardiovasc Dis.

[21] Döring M, Sommer P, Rolf S, Lucas J, Breithardt OA, Hindricks G, Richter S (2015). Sensor-based electromagnetic navigation to facilitate implantation of left ventricular leads in cardiac resynchronization therapy. J Cardiovasc Electrophysiol.

[22] (2022). The Texas Heart Institute: Clinical trial to evaluate a new system for patients with irregular heartbeats. https://www.texasheart.org/clinical-trial-to-evaluate-a-new-system-for-patients-with-irregular-heartbeats/..

[23] Fang F, Jie ZY, Xia LX, Ming L, Zhan M, Fen GS, Cheuk-Man Y (2015). Cardiac resynchronisation therapy and heart failure: Persepctive from 5P medicine. Card Fail Rev.

[24] de Abreu RC, Fernandes H, da Costa Martins PA, Sahoo S, Emanueli C, Ferreira L (2020). Native and bioengineered extracellular vesicles for cardiovascular therapeutics. Nat Rev Cardiol.

[25] Butcher C, Mareev Y, Markides V, Mason M, Wong T, Cleland JG (2015). Cardiac resynchronization therapy update: evolving indications, expanding benefit?. Curr Cardiol Rep.

[26] Cohen IG, Gerke S, Kramer DB (2020). Ethical and legal implications of remote monitoring of medical devices. Milbank Q.

[27] Ullah W, Zahid S, Zaidi SR (2021). Predictors of permanent pacemaker implantation in patients undergoing transcatheter aortic valve replacement - A systematic review and meta-analysis. J Am Heart Assoc.

[28] Mehra R, Ziegler P, Sarkar S, Ritscher D, Warman E (2006). Management of atrial tachyarrhythmias. Rhythm control using implantable devices. IEEE Eng Med Biol Mag.

[29] Spentzou G, Mayne K, Fulton H, McLeod K (2019). Virtual clinics for follow-up of pacemakers and implantable cardioverter defibrillators in children. Cardiol Young.

[30] Cribier A, Eltchaninoff H, Tron C (2004). Early experience with percutaneous transcatheter implantation of heart valve prosthesis for the treatment of end-stage inoperable patients with calcific aortic stenosis. J Am Coll Cardiol.

[31] Tinica G, Tarus A, Enache M, Artene B, Rotaru I, Bacusca A, Burlacu A (2020). Infective endocarditis after TAVI: a meta-analysis and systematic review of epidemiology, risk factors and clinical consequences. Rev Cardiovasc Med.

[32] Williams ML, Flynn CD, Mamo AA (2020). Long-term outcomes of sutureless and rapid-deployment aortic valve replacement: a systematic review and meta-analysis. Ann Cardiothorac Surg.

[33] Zheng Q, Tang Q, Wang ZL, Li Z (2021). Self-powered cardiovascular electronic devices and systems. Nat Rev Cardiol.

[34] Sperzel J, Hamm C, Hain A (2018). Nanostim-leadless pacemaker. Herzschrittmacherther Elektrophysiol.

[35] Gutruf P, Yin RT, Lee KB (2019). Wireless, battery-free, fully implantable multimodal and multisite pacemakers for applications in small animal models. Nat Commun.

[36] Tarakji KG, Zaidi AM, Zweibel SL (2021). Performance of first pacemaker to use smart device app for remote monitoring. Heart Rhythm O2.

[37] El Hamriti M, Imnadze G, Sohns C, Sommer P (2020). [Smart decisions in rhythmology : What should we know? What should be do? What do we still have to learn?]. Herzschrittmacherther Elektrophysiol.

